# Dislocation-enhanced experimental-scale vacancy loop formation in hcp Zirconium in one single collision cascade

**DOI:** 10.1038/srep21034

**Published:** 2016-02-12

**Authors:** Wei Zhou, Jiting Tian, Jian Zheng, Jianming Xue, Shuming Peng

**Affiliations:** 1 Institute of Nuclear Physics and Chemistry, China Academy of Engineering Physics, Mianyang, People’s Republic of China, 621999; 2 State Key Laboratory of Nuclear Physics and Technology, School of Physics, Peking University, Beijing, People’s Republic of China, 100871; 3 Center for Applied Physics and Technology, Peking University, Beijing, People’s Republic of China, 100871

## Abstract

Large defects are the main factor leading to the degradation of material properties under irradiation environments. It is commonly assumed that the large defects are mainly formed through cluster growth under continuous irradiations. Besides this mechanism, recent experiments and simulations show that sometimes an individual ion can also directly create a large defect. Here we report a novel mechanism for the formation of the large defects, as discovered by our Molecular Dynamics (MD) simulations of the collision cascades in hcp Zirconium (Zr): a pre-existing edge dislocation (ED) can significantly promote the nucleation of the vacancy clusters, and even facilitate the direct formation of an experimental-scale large vacancy loop (about 3 nm) in only one single displacement cascade. This dislocation-related mechanism may be the key for understanding the experimental results in the low-dose irradiated Zr where the high-density large dislocation loops are observed but difficult to be explained by the two mechanisms mentioned above. Considering that intrinsic dislocations exist in nearly all crystalline materials, our results provide a significant concept: pre-existing dislocations have a strong influence on the primary damage production, and taking them into account is indispensable for assessing and improving the material’s irradiation-resistance.

Designing materials which are adequate for extreme irradiation environments in future nuclear energy systems remains still a huge challenge in materials science^1^,^2^,^3^. Comprehensive and thorough understanding of the damage process in irradiated materials is the key for assessing and improving the material performance. It is well-known that the degradation (swelling, hardening, embrittlement, etc) of material engineering properties is mainly caused by defects, especially by the large defects (voids, dislocation loops, etc)^4^,^5^. Therefore, knowledge of the formation and evolution of the large clusters, plays a key role in understanding the material behaviour under irradiation environments.

It is commonly assumed that the large defects are mainly formed through cluster growth due to the inter-cascade interactions under continuous irradiations. More specially, the primary knocked-on atoms (PKAs) initiated by the incident energetic ions can produce some point defects and small clusters along their trajectories. Then, these small defects in a local zone can migrate and combine with each other, to grow and transform into observable complex defects, such as voids and dislocation loops^4^,^5^. However, this traditional description is difficult to explain the defects observed in very low-dose irradiation experiments, where the point defects or small clusters were not dense enough^6^. For example, irradiation experiments in hcp Zr, which is one of the most important materials in nuclear reactors, have shown that high-density (1.35/100 *nm*^2^) observable dislocation loops (2~3 nm) can be formed at very low dose (0.008 dpa)^7^. Under such low dose, the produced point defects and small clusters are insufficient to lead to such dense distribution of large clusters.

Another possible mechanism for large defect formation is that, while a PKA mostly produces only small defects, occasionally it is also able to create an experimental-scale defect cluster in only one individual collision cascade. Stoller termed this kind of the extreme irradiation effects as “rare events”, and suggested that in low-dose experiments where aggregation of point defects is difficult, significant rare cascade events are the key for understanding the damage production^6^. A typical example of this kind of the rare events is that Soneda and coworkers^8^ have found that in one of 100 MD simulation runs of 50 keV cascades in defect-free alpha-Fe, a large vacancy loop containing 153 vacancies is directly created by a single PKA, while the defect clusters in the other 99 simulations are much smaller (containing less than 50 point defects). Very recently, this kind of rare events are observed in self-ion irradiation experiments of W at cryogenic temperature, and the occurring frequency is in perfect agreement with MD simulations (both less than 1% per ion)^9^. However, this low-probability mechanism should not be the main explanation for the experimental results in Zr^7^. This is because the experimental defect yield at 0.008 dpa calculated by the authors is as high as 0.27^7^, which means that on the average an incident ion can create 0.27 loops, while the probability of the rare events is only about 1% per ion. Additionally, MD simulations show that defect clusters formed in a 20 keV cascade in a single crystal of hcp Zr always contain less than 50 point defects^10^, and no simulations for higher-energy PKAs have been performed, implying that whether the rare events could occur in hcp Zr is still unclear.

We suggest that taking the intrinsic dislocations into account in MD simulations of collision cascades might provide more reliable explanations. This idea is inspired by the previous works which show that some kind of pre-existing microstructures such as grain boundaries can promote the vacancy production and induce even larger defects than in the bulk^11^,^12^,^13^. Actually, Voskoboinikov has reported that a pre-exiting edge dislocation (ED) in fcc aluminium (Al) can greatly increase the number of the survived vacancies at high temperatures, and facilitate the formation of a stacking fault tetrahedra (SFT)^14^. However, the microscopic process of the interaction between the ED and the collision cascade is still blurry, and the relationship between this kind of the microstructure’s enhancing effect and the experimental results needs more explorations.

In this paper, we report the results of our simulations of 5–20 keV collision cascades close to an 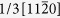 ED in hcp Zr using empirical-potential MD method. We find that the pre-exiting ED is able to significantly promote the nucleation of the vacancy clusters, and a single 20 keV PKA can even directly create an experimental-scale vacancy loop (~3 nm) with a high probability. With an additional heat annealing, this kind of 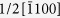 vacancy loop can be transformed into a 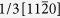 dislocation loop, in good agreement with experimental observations^15^,^16^,^17^. To the best of our knowledge, this is the first time that the whole damage process in hcp-Zr from a PKA-initiated collision cascade to the formation of an experimental-scale 1/3^11^,^12^,^13^,^14^,^15^,^16^,^17^,^18^,^19^,^20^ vacancy loop is continuously observed by simulations. Our results, combined with the traditional “diffusion and aggregation” mechanism, offer a reliable explanation for the formation of high-density large defects in the low-dose irradiation experiments of hcp Zr^7^.

## Results

### Simulated model system

**Figure 1:**
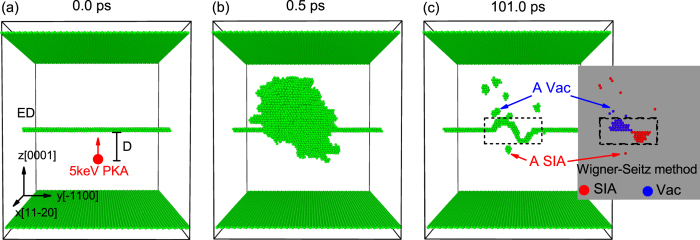
Temporal evolution of a typical simulated collision event close to the ED in hcp Zr at 300 K. Only the irregular atoms are displayed as green solid spheres (according to the a-CNA method), including the outmost rigid layers, the ED and the disordered atoms due to atomic collisions. Note that for a point defect, all the neighbour atoms are regarded as disordered atoms for their irregular coordination numbers. The inset in (**c**) shows the corresponding distribution of the point defects certified by the Wigner-Seitz method. The defects in the dashed box are actually induced by the rearrangement of the atoms near the dislocation, thus we have not counted them as surviving defects.

We display our model system for the 5 keV cascade events in Fig. 1. An 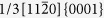 ED along the Y axis (crystal orientation of 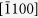) is created in a hcp Zr crystal using the “strain and spline” approach (see Method for details). When simulating the collision events, we set the atoms in the top and the bottom layer along the Z direction [0001] to be rigid, avoiding the movement of the whole sample. We choose an atom within a 2 × 2 nm^2^ area under the dislocation as a PKA and give it a suitable velocity along the +Z direction (Fig. 1(a)). Then this PKA is able to induce a displacement cascade close to the ED (Fig. 1(b)). Usually the cascade peaks at 0.5 ps, and almost cools down after 20 ps, but here we run our simulations for a total of 101 ps to allow all the possible interaction events in the primary stage to happen. The adaptive common neighbor analysis (a-CNA) method^18^ is employed to display the irregular atoms (including the rigid layers, the dislocation structure and the irradiation-induced defects) while the Wigner-Seitz method is applied to analyze the type and number of the point defects, *i.e.* self-interstitial atom (SIA) and vacancy (Vac). We stress that a cascade will lead to a serious rearrangement of the atoms near the dislocation. To avoid regarding the rearranged atoms as defects, we only count the defects not connected with the dislocation (the defects outside the dashed box in Fig. 1(c)). This method for counting defects has previously been used in the research on cascades near a grain boundary^11^. To obtain detailed and systematic knowledge on the interactions between the cascade and the ED, we use 4 different distances (*D* = 1.0, 3.0, 5.0 or 7.0 nm) for the 5 keV PKA away from the ED. For each distance, 10 simulation runs with different random-selected PKA horizontal locations are performed to get statistical data. (Details for the simulated system can be found in the Method section.)

### Statistics of defect production

**Figure 2:**
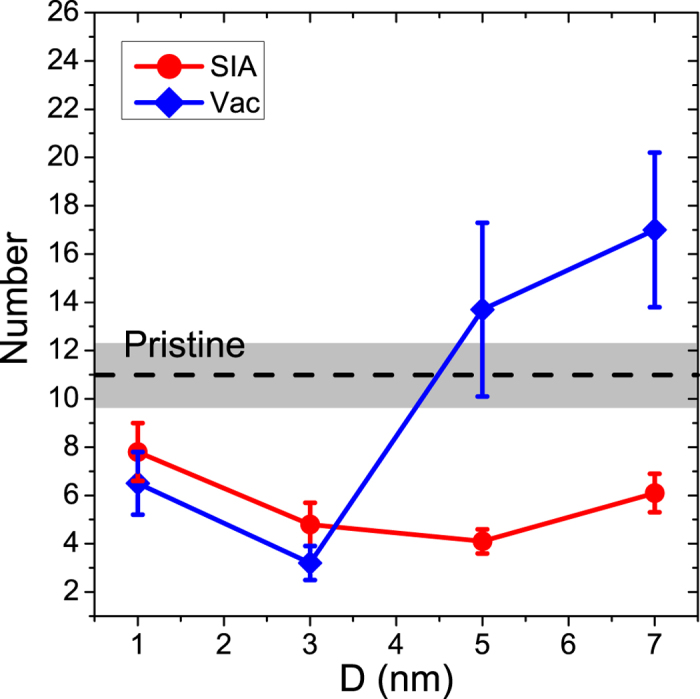
The statistical results of the surviving defects not connected with the ED in hcp Zr induced by 5 keV PKAs at 300 K as a function of the initial PKA distance D from the ED. Each solid line is an average of 10 simulation runs, and the error is the standard error of the mean. The black dashed line with the grey zone represents the result (11.0 ± 1.4) of simulations in the pristine hcp Zr.

Firstly, we take the results of the simulations of the 5 keV cascades at 300 K as a typical example to provide a general understanding on the ED’s effects on the primary damage production. Figure 2 shows the numbers of the surviving defects after the cascade relaxation for different PKA distances. It is clear that the number of the surviving SIAs decreases with *D* at first and then increases at larger *D*. This phenomenon can be easily understood by the overlap between the cascade core and the ED. As the distance increases, the overlap region between the cascade and the ED increases at first, peaks at around *D* = 5.0 nm and then decreases. Thus the absorption effect of the ED for SIAs becomes stronger at first and then weaker. We can expect that when the distance is large enough, there will be no overlap and the defect production will be close to that in the bulk case (grey region in Fig. 2). A similar variation trend of the SIA number along with the PKA distance has also been observed in the previous simulations of cascades near a grain boundary in Cu^11^. However, the vacancy production seems very different from that of SIA: while the variation trend still exhibits an approximate U shape, the number of the vacancies changes drastically along with the distance. The huge standard errors of the two data points above the black dashed line imply that some “special events” have emerged in the 10 simulation runs for *D* = 5.0 nm or *D* = 7.0 nm.

**Figure 3:**
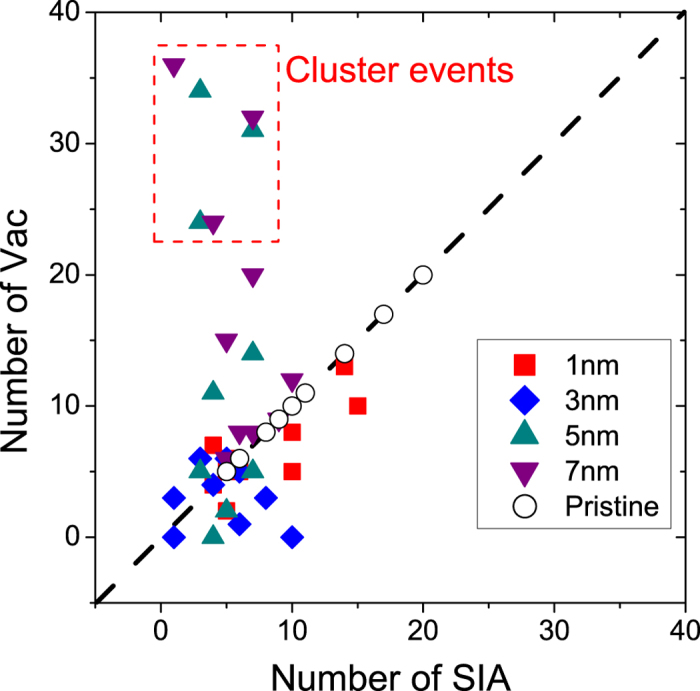
The number of vacancies and SIAs formed in 5 keV collision cascades in pristine hcp Zr and in hcp Zr with an ED at 300 K. The dashed line indicates the diagonal. Each point corresponds to a simulation run.

To figure out where the large numbers of the vacancies at *D* = 5.0 nm and *D* = 7.0 nm come from, we display the defect number of each collision event in Fig. 3. As the number of the SIAs and the vacancies formed in the pristine Zr is equal, the corresponding data points should locate on the diagonal line(see white circles in Fig. 3). While most data points in Fig. 3 are close to the diagonal, several points for *D* = 5.0 nm and *D* = 7.0 nm stay far away from it. Actually these points are critically related to the special events mentioned above. A closer look at the snapshots of the cascade evolution shows that large vacancy clusters are formed in these simulation runs. We term them “cluster events” and will analyze them in the next section.

### Formation of large vacancy clusters

By counting the cluster sizes in the cluster events at *D* = 5.0 nm and *D* = 7.0 nm, we find that large vacancy clusters containing 20–35 vacancies have been formed. In contrast, without the existence of the ED, the largest vacancy cluster formed in the 5 keV cascades in the pristine crystal only contains 14 vacancies (Table 1). Gao *et al.* have shown a similar result that the vacancy clusters produced in the 5 keV cascades in defect-free hcp Zr contain less than 10 vacancies^10^. A closer observation reveals that the large clusters usually are 3D disordered zones with vacancies being “dissolved” therein. With an additional annealing of 50 ps at 600 K, these vacancy clusters usually change into more regular faulted loops with 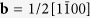. This result is in good agreement with the previous simulations in Zr crystals^10^.

**Figure 4:**
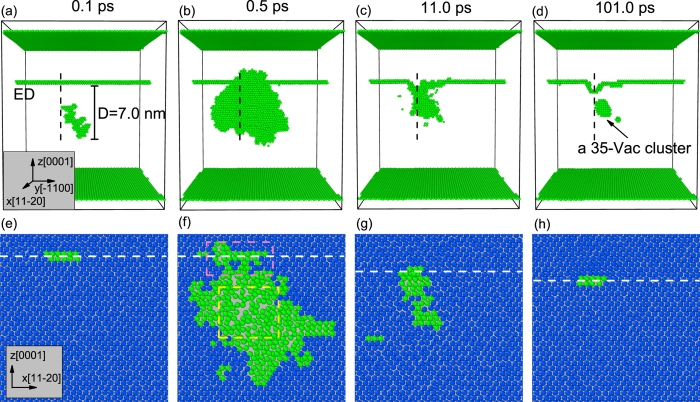
Temporal evolution of a typical cluster event induced by a 5 keV PKA at *D* = 7.0 nm at 300 K. The top frames (**a**–**d**) show the development of the displacement cascade and the bottom frames (**e**–**h**) display the atomic rearrangement of one layer in the (1–100) plane corresponding to a slice indicated by the black vertical dashed line in the top frames. In (**a**–**d**), only the irregular atoms are displayed as green solid spheres (according to the a-CNA method). In (**e**–**h**), the irregular atoms are displayed as green solid spheres while the crystalline atoms as blue ones. The white horizontal dashed lines in (**e**–**h**) indicate the boundary between the denser and the looser mode. The pink box indicates the cascade edge which is crowded with SIAs, and the yellow one corresponds to the cascade core where vacancies are dominated.

To analyze how the large vacancy clusters are formed, we demonstrate the temporal evolution of a typical cluster event for *D* = 7.0 nm in Fig. 4. The top frames show the development of the displacement cascade and the bottom frames display the atomic rearrangement of one layer in the 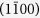 plane corresponding to a slice indicated by the black vertical dashed line in the top frames. From Fig. 4 we can see that an energetic PKA introduces a local disordered region with many displaced atoms. As the cascade cools down, most atoms rearrange themselves into the surrounding lattice sites. This kind of “melt and recrystallization” process has been demonstrated in many previous simulations of cascades in metals^6^,^19^,^20^,^21^,^22^,^23^. It is well-known that in the recrystallization process, disordered atoms prefer to locate themselves following the surrounding lattice sites of the ordered atoms^24^. During the relaxation of a cascade in a perfect single crystal, melt atoms have only one structure mode to follow. However, for atoms close to the ED in the present atomic model, as the upper half-crystal beyond the slip plane (0001) contains two more 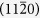 layers of atoms than the lower one within the same width along the X 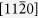 axis, two different modes are alternative: the denser mode above the slip plane and the looser one below it. For cluster events at *D* = 5.0 nm and *D* = 7.0 nm, the cascade region can be asymmetrical around the ED. More specifically, the cascade zone can just “hang” on the dislocation line (see Fig. 4(b)). In this case, the upper part of the cascade beyond the ED will cool down faster than the lower one does, making the hot atoms near the ED rearrange themselves following the denser mode by absorbing SIAs (see Fig. 4(f–h)). As a result, a segment of the ED climbs downward locally and many vacancies are left to form a large cluster, as shown in Fig. 4(d). In addition, the fact that a displacement cascade often pushes SIAs outward to its edge, which has been shown by many simulations^19^,^21^, will facilitate the ED’s absorption effect for SIAs in the cluster events (see the pink dashed box in Fig. 4(f)).

We emphasize that not all of the simulation runs for *D* = 5.0 nm and *D* = 7.0 nm have produced large vacancy clusters (six of the twenty runs in our study, see Fig. 3). Comparing the cluster events and the normal ones at the same initial PKA distance, we find that the development of a cascade is a stochastic process, and the shape of the cascade region varies drastically among different runs. Only when the cascade zone is heavily asymmetrical around the ED can the large vacancy cluster be created. For *D* = 3.0 nm, the cascade region is usually symmetrical around the ED, thus no obvious selective absorbtion for SIAs or vacancies emerges.

We have expanded our simulations to 10 and 15 keV collision events. It is found that the PKAs with the higher energies lead to larger disordered regions and more surviving point defects. Sometimes even subcascades are formed. Similar with the 5 keV collision cascades, when the cascade zone (or a part of the cascade zone, when subcascades are formed) just hangs on the ED, large vacancy clusters can be created, accompanied with the local climb behaviour of the ED (see Supplementary Fig. S3). As displayed in Table 1, the largest vacancy clusters created by the 10 and 15 keV PKAs in the sample with an ED contain 73 and 85 vacancies, respectively, while those in the pristine sample are much smaller (9 and 25 vacancies, respectively). The result indicates that the large vacancy cluster formation enhanced by the pre-existing ED is a general phenomenon independent of the PKA’s energy.

### Formation of experimental-scale dislocation loops

Finally, we change the simulation condition to the normal irradiation environment in nuclear reactors, *i.e.* 20 keV PKA and 600 K^15^. 10 simulation runs are performed for the cascade induced by a PKA initially locating at *D* = 7.0 nm under the ED in a hcp Zr crystal. Besides, cascades in the pristine hcp Zr under the same irradiation condition are also simulated. We find that, while the largest cluster formed in the defect-free crystal has only 32 vacancies, which is close to Gao *et al.*’s result (24 vacancies)^23^, the ED in hcp Zr is capable of contributing for the formation of large clusters containing up to 92 vacancies (Table 1).

**Figure 5:**
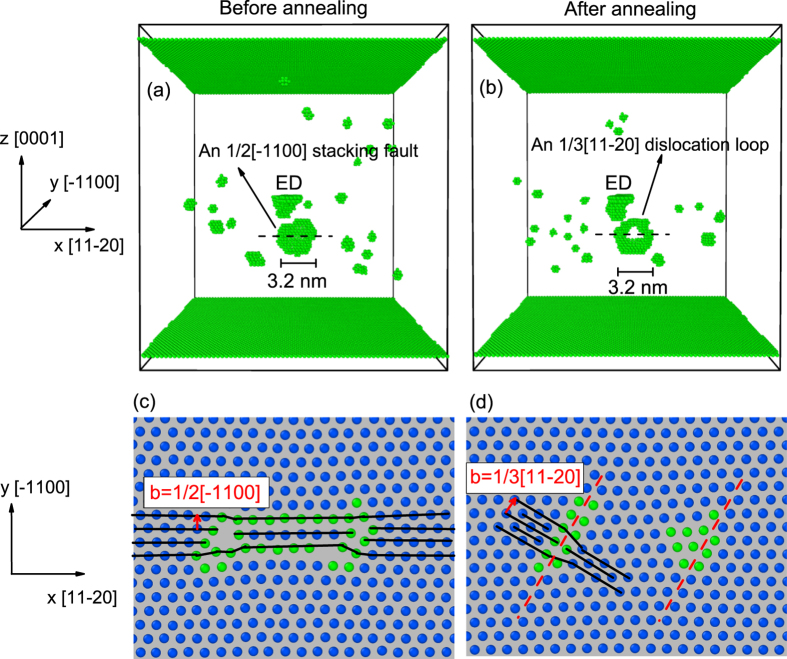
Atomic configuration of the sample in which a 92-vacancy cluster has been formed after a 20 keV cascade event at 600 K. The top frames (**a**,**b**) show the atomic configuration of the whole sample and the bottom frames (**c**,**d**) depict the atomic configuration of one layer in the basal plane (0001) corresponding to a slice indicated by the black horizontal dashed line in the top frames. In (**a**,**b**) only the irregular atoms are displayed as green spheres (according to the a-CNA method). In (**c**,**d**) the irregular atoms are displayed as green solid spheres while the crystalline atoms as blue ones. In (**d**) the red dashed lines indicate the slip planes of the dislocations.

Figure 5 demonstrates the atomic configuration of the sample (top (a) and (b)), in which a 92-vacancy cluster has been formed, as well as the atomic configuration of one layer (bottom (c) and (d)) in the basal plane (0001) corresponding to a slice indicated by the black horizontal dashed line in the top frames. This vacancy cluster directly created in the cascade has a size of 10 *a*_0_ (~3.2 nm), situated in the first-order prism plane 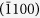 (see Fig. 5(a)). A closer look at the one-layer atomic configuration shows that this cluster has been collapsed into an E-type extrinsic stacking fault (Fig. 5(c)). Similar structure has also been found in the previous atomic models of vacancy clusters in hcp Zr^25^. However, several researchers have suggested that this kind of stacking fault is metastable and can be transformed into an 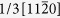 dislocation loop through a 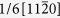 shear^25^,^26^,^27^. To realize this transformation, we use a short-time high-temperature annealing to model the long-term relaxation of the sample under room temperature^28^. In detail, we heat the whole sample for an additional 100 ps at 1000 K, and then cool it down to 300 K. The final configurations are depicted in Fig. 5(b,d). It is obvious that the stacking fault has been changed into a dislocation loop (see Fig. 5(b)). The one-layer atomic arrangement shows that the collapsed structure has been transformed into two dislocation cores, each with a 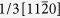 Burgers vector. The final dislocation loop mainly lies on the first-order prism plane 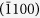, and is large enough to be observable under TEM in experiments^7^.

## Discussions

We have performed MD simulations of 5–20 keV displacement cascades in hcp Zr with the the presence of a pre-existing ED. Our results can explain the experimental finding that high-density dislocation loops with a size of 2–3 nm have been observed in hcp Zr irradiated by 1 MeV Kr^2+^ ions at 0.008 dpa^7^. This experimental phenomenon is difficult to be elucidated alone by the traditional “diffusion and aggregation” framework, because under such low dose, the produced small defects are insufficient to lead to such dense distribution of large clusters. The phenomenon is also impossible to be explained alone by the rare events of in-cascade formation of large clusters in a defect-free crystal, because the experimental value of the defect yield (0.27) is much larger than the occurring frequency of the rare events (usually less than 1% per ion). On the contrary, our simulations provide a high-probability (larger than 10% per cascade in our simulations) mechanism that an intrinsic ED is able to increase the number of the survived vacancies, promote the nucleation of the vacancy clusters, and even directly create an experimental-scale vacancy loop in only one collision cascade. Using SRIM 2008^29^, we find that an 1 MeV Kr^2+^ ion usually introduces hundreds of PKAs and cascades, and the whole cascade region can expand as large as around 100 × 100 × 400 nm^3^. This result implying that the probability of interactions between the cascade region and the pre-existing ED should not be ignored. Besides, our finding is not contrary to the “diffusion and aggregation” description, as the nucleated vacancy clusters near the ED can still absorb the diffusing point defects or small clusters to grow into larger defects. Consequently, the combination of the ED-enhance effect and the “diffusion and aggregation” mechanism may be a reliable explanation for the high-density loops, especially the high defect yield found in the low-dose experiments^7^.

In recent years, intense attention has been paid to the pre-existing defect’s influence on the damage process and the irradiation tolerance in materials. While point defects (0D defect)^30^, grain boundaries (2D)^11^,^12^,^13^, and nanovoids (3D)^31^ have been studied, few research on dislocations (1D) have been reported. Actually, there are three reasons supporting the necessity for studying dislocation’s effects on radiation damage. Firstly, as one of the most important defects in crystals, dislocations universally exit in most metals, especially in cold-rolled metallic materials. More specially, even in well-annealed metal crystals, the dislocation network density is on the order of ~10^11^ *m*^−2^ (much higher in cold-rolled materials, ~10^14^ *m*^−2^)^26^, implying that dislocation’s effects should be adequately considered. Secondly, experiments have shown that cold-worked and heat-annealed metallic materials behave quite differently under low-dose irradiations (see^32^ and references cited therein), implying that the pre-exiting dislocation networks in the samples play a significant role in the damage process. Finally, MD simulations in Cu have shown that grain boundaries, another kind of widely-existed intrinsic defects in crystals, can exhibit an obvious absorption preference for the interstitials and promote the formation of a stacking fault tetrahedra (SFT)^11^. Similar with grain boundaries, dislocations can also work as sinks, thus we can expect that simulations considering the presence of the pre-exiting dislocations in the samples may provide some novel results.

Our simulations, indeed, have shown that dislocations have a strong influence on the primary damage production. More specially, a pre-exiting ED can 1) increase the number of the survived vacancies, 2) promote the nucleation of the vacancy clusters, and 3) even help to directly create an experimental-scale vacancy loop in only one individual collision cascade. Considering that the degradation of material engineering properties in irradiation environments mainly stems from the large defect clusters (voids, dislocations loops *et al.*) instead of point defects, we suggest that heat-annealed and low-stress materials should be more tolerant to radiation damage.

## Conclusion

We have simulated 5–20 keV collision cascades in hcp Zr containing an ED with classical MD method. We find that large vacancy clusters can be generated when the cascade zone (or a part of the cascade zone, when subcascades are formed) is heavily asymmetrical around the ED. The largest vacancy cluster emerging in the present simulations is large enough (~3.2 nm) to be observed in experiments. We advise that this kind of ED-enhanced formation of the large vacancy clusters may be closely related to the dislocation loops (2–3 nm) found in the low-dose irradiation experiments of hcp Zr. Our work suggests that the pre-existing dislocations in materials have a strong influence on the primary defect production, and taking them into account is indispensable for assessing and improving irradiation-resistance in metallic materials.

## Method

We have performed all the MD simulations with the LAMMPS code^33^. It is well-known that accuracy of the empirical potential plays a key role in classical MD simulations. Here we choose the embedded atom method (EAM) potential for hcp Zr developed by Mendelev and Ackland^34^ to model atomic interactions. This potential has been proved to be capable of providing a reliable description of the dislocation structure^35^ and collision cascades^36^ in hcp Zr.

An 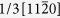 ED along the Y axis (crystal orientation of 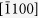) is created in a hcp Zr crystal using the “strain and spline” approach (see reference^37^ for details). Briefly, two half-crystals with different lengthes (N *a*_0_ and N-1 *a*_0_) are strained to the same length (N-0.5 *a*_0_) along the X [11-20] axis. Then we spline the two half-crystals along the dislocation slip plane (0001). After relaxation and quenching, an edge dislocation with a Burgers vector 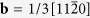 will be created at the center of the sample. The final displacement field is displayed in Supplementary Fig. S1(a). We calculate the disregistry *D*(*x*), defined by the displacement difference between the atoms in the plane just above and just below the slip plane, and the dislocation density 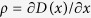, defined by the derivative of the disregistry *D*(*x*). The result shows that the dislocation core contains two dissociated lines with a distance of 4 *a*_0_ (Supplementary Fig. S1(b)), in excellent agreement with the previous atomic model of an ED in hcp Zr^35^. The potential map of the sample also depicts the same dissociation distance (Supplementary Fig. S1(c)). Note that the ED created here prefers to slip on the basal plane {0001}, instead of the prism one 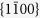, while the latter case is much more dominant in hcp Zr crystal^26^. However, in this work we are mainly interested in the dislocation’s local climb behaviour during the primary damage stage rather than slip, thus the choice of the preferred slip plane should not has a significant effect on the results. Actually, we have performed test simulations using an 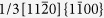 edge dislocation, and the results reveal no notable differences (Supplementary Fig. S2).

The Zr crystal is as large as 180 × 180 × 180 Å^3^ (containing 258,304 atoms) or 230 × 230 × 230 Å^3^ (containing 558,600 atoms), depending on the PKA’s kinetic energy (5 keV or 10–20 keV). The sample sizes are chosen to ensure that few atomic collisions happen at boundaries. The X and Y boundaries of the system are periodic and the Z boundaries free. Before initiating a collision event, we relax the whole sample at 300 K (or 600 K) for 10 ps using a Nose-Hoover thermostat^38^. Then the atoms in the top and the bottom layer along the Z direction [0001] are set to be rigid, avoiding for the movement of the whole sample. Temperature scaling of 300 K (or 600 K) with the Berendsen method^39^ is applied at the atoms in the outmost two layers along all the three directions to dissipate the heat. A variable time step is used according to the fastest moving particles in the system. Usually the cascade peaks at 0.5 ps, and almost cools down after 20 ps, but here we run our simulations for a total of 101 ps to allow all the possible interaction events in the primary stage to happen. To obtain detailed and systematic knowledge on the interactions between the cascade and the ED, we use 4 different distances for the 5–15 keV PKA away from the ED. For each distance, the ED’s position is adjusted to make sure that the core of the cascade is at the center of the sample, and 10 simulation runs with different PKA horizontal locations randomly chosen from a 2 × 2 nm^2^ area under the ED are performed to get statistical data. Although it is well-known that electronic excitations should be carefully addressed in simulations of keV collision cascades, here we ignore this effect for the sake of comparison with the previous atomic simulations.

The adaptive common neighbor analysis (a-CNA) method^18^ is employed to display the irregular atoms (including the rigid layers, the dislocation structure and the irradiation-induced defects) while the Wigner-Seitz method is applied to analyze the type and number of point defects, *i.e.* self-interstitial atom (SIA) and vacancy (Vac). Both of the two methods have been implanted into the visualization tool OVITO^40^. We stress that a cascade will lead to a serious rearrangement of the atoms near the dislocation. To avoid regarding the rearranged atoms as defects, we only count the defects not connected with the dislocation (the defects outside the dashed box in Fig. 1(c)). This method for counting defects has previously been used in the research on cascades near a grain boundary^11^.

## Additional Information

**How to cite this article**: Zhou, W. *et al.* Dislocation-enhanced experimental-scale vacancy loop formation in hcp Zirconium in one single collision cascade. *Sci. Rep.* **6**, 21034; doi: 10.1038/srep21034 (2016).

## Supplementary Material

Supplementary Movie S1

Supplementary Information

## Figures and Tables

**Table 1:** The number of vacancies in the largest vacancy cluster created in the samples with or without a pre-existing ED in different simulation conditions.

Simulation condition	The number of vacancies in the largest cluster	
	Without ED	With ED
5 keV, 300 K	14	35
10 keV, 300 K	9	73
15 keV, 300 K	25	85
20 keV, 600 K	32	92

## References

[b1] Wirth B., Nordlund K., Whyte D. & Xu D. Fusion materials modeling: Challenges and opportunities. MRS bulletin 36, 216–222 (2011).

[b2] Zinkle S. J. & Was G. Materials challenges in nuclear energy. Acta Mater. 61, 735–758 (2013).

[b3] Zinkle S. J. & Snead L. L. Designing Radiation Resistance in Materials for Fusion Energy. Annu. Rev. Mater. Res. 44, 241–267 (2014).

[b4] Was G. S. Fundamentals of radiation materials science: metals and alloys (Springer Science & Business Media, 2007).

[b5] Uberuaga B., Hoagland R., Voter A. & Valone S. Direct transformation of vacancy voids to stacking fault tetrahedra. Phys. Rev. Lett. 99, 135501 (2007). 17930607 10.1103/PhysRevLett.99.135501

[b6] Stoller R. Comprehensive Nuclear Materials vol. 1 (Elsevier, 2012).

[b7] Idrees Y., Yao Z., Kirk M. & Daymond M. *In situ* study of defect accumulation in zirconium under heavy ion irradiation. J. Nucl. Mater. 433, 95–107 (2013).

[b8] Soneda N., Ishino S. & De la Rubia T. D. Vacancy loop formation by’cascade collapse’in a-Fe: A molecular dynamics study of 50keV cascades. Philos. Mag. Lett. 81, 649–659 (2001).

[b9] Yi X. *et al.* Direct observation of size scaling and elastic interaction between nano-scale defects in collision cascades. Eur. Phys. Lett. 110, 36001 (2015).

[b10] Gao F., Bacon D., Howe L. & So C. Temperature-dependence of defect creation and clustering by displacement cascades in *α*-zirconium. J. Nucl. Mater. 294, 288–298 (2001).

[b11] Bai X.-M., Voter A. F., Hoagland R. G., Nastasi M. & Uberuaga B. P. Efficient annealing of radiation damage near grain boundaries via interstitial emission. Science 327, 1631–1634 (2010). 20339070 10.1126/science.1183723

[b12] Bai X.-M. & Uberuaga B. P. The Influence of Grain Boundaries on Radiation-Induced Point Defect Production in Materials: A Review of Atomistic Studies. JOM 65, 360–373 (2013).

[b13] Uberuaga B. P., Vernon L. J., Martinez E. & Voter A. F. The relationship between grain boundary structure, defect mobility, and grain boundary sink efficiency. Sci. Rep. 5, 9095, 10.1038/srep09095 (2015). 25766999 PMC4357896

[b14] Voskoboinikov R. E. MD simulations of collision cascades in the vicinity of a screw dislocation in aluminium. Nucl. Instrum. Methods Phys. Res., Sect. B 303, 104–107 (2013).

[b15] Onimus F. & Béchade J. Comprehensive Nuclear Materials vol. 4 (Elsevier, 2012).

[b16] Griffiths M. A review of microstructure evolution in zirconium alloys during irradiation. J. Nucl. Mater. 159, 190–218 (1988).

[b17] Griffiths M. Evolution of microstructure in hcp metals during irradiation. J. Nucl. Mater. 205, 225–241 (1993).

[b18] Stukowski A. Structure identification methods for atomistic simulations of crystalline materials. Modelling Simul. Mater. Sci. Eng. 20, 045021 (2012).

[b19] De La Rubia T. D., Averback R., Benedek R. & King W. Role of thermal spikes in energetic displacement cascades. Phys. Rev. Lett. 59, 1930 (1987). 10035371 10.1103/PhysRevLett.59.1930

[b20] De La Rubia T. D. & Guinan M. New mechanism of defect production in metals: A molecular-dynamics study of interstitial-dislocation-loop formation in high-energy displacement cascades. Phys. Rev. Lett. 66, 2766 (1991). 10043611 10.1103/PhysRevLett.66.2766

[b21] Nordlund K. *et al.* Defect production in collision cascades in elemental semiconductors and fcc metals. Phys. Rev. B 57, 7556 (1998).

[b22] Caturla M. *et al.* Comparative study of radiation damage accumulation in Cu and Fe. J. Nucl. Mater. 276, 13–21 (2000).

[b23] Bacon D., Gao F. & Osetsky Y. N. The primary damage state in fcc, bcc and hcp metals as seen in molecular dynamics simulations. J. Nucl. Mater. 276, 1–12 (2000).

[b24] Nordlund K. & Averback R. Inverse Kirkendall mixing in collision cascades. Phys. Rev. B 59, 20 (1999).

[b25] De Diego N., Osetsky Y. N. & Bacon D. Structure and properties of vacancy and interstitial clusters in *α*-zirconium. J. Nucl. Mater. 374, 87–94 (2008).

[b26] Hull D. & Bacon D. J. Introduction to dislocations vol. 37 (Elsevier, 2011).

[b27] Varvenne C., Mackain O. & Clouet E. Vacancy clustering in zirconium: An atomic-scale study. Acta Mater. 78, 65–77 (2014).

[b28] Lau D. *et al.* Abrupt stress induced transformation in amorphous carbon films with a highly conductive transition phase. Phys. Rev. Lett. 100, 176101 (2008). 18518310 10.1103/PhysRevLett.100.176101

[b29] Ziegler J. *SRIM 2008*. (2008) Available at: http://www.srim.org/SRIM/SRIMLEGL.htm. (Accessed: 15th October 2015).

[b30] Nordlund K. & Averback R. Point defect movement and annealing in collision cascades. Phys. Rev. B 56, 2421 (1997).

[b31] Chen Y. *et al.* Damage-tolerant nanotwinned metals with nanovoids under radiation environments. Nat. Commun. 6, 7036, 10.1038/ncomms8036 (2015). 25906997 PMC4421808

[b32] Garner F. Irradiation performance of cladding and structural steels in liquid metal reactors. In Mater. Sci. Technol. (Wiley Online Library, 2006).

[b33] Plimpton S. Fast parallel algorithms for short-range molecular dynamics. J. Comput. Phys. 117, 1–19 (1995).

[b34] Mendelev M. I. & Ackland G. J. Development of an interatomic potential for the simulation of phase transformations in zirconium. Philos. Mag. Lett. 87, 349–359 (2007).

[b35] Khater H. & Bacon D. Dislocation core structure and dynamics in two atomic models of *α*-zirconium. Acta Mater. 58, 2978–2987 (2010).

[b36] Di S. *et al.* Dislocation-accelerated void formation under irradiation in zirconium. Acta Mater. 82, 94–99 (2015).

[b37] Osetsky Y. N. & Bacon D. J. An atomic-level model for studying the dynamics of edge dislocations in metals. Modelling Simul. Mater. Sci. Eng. 11, 427 (2003).

[b38] Martyna G. J., Klein M. L. & Tuckerman M. Nosé–Hoover chains: the canonical ensemble via continuous dynamics. J. Chem. Phys. 97, 2635–2643 (1992).

[b39] Berendsen H. J., Postma J. P. M., van Gunsteren W. F., DiNola A. & Haak J. Molecular dynamics with coupling to an external bath. J. Chem. Phys. 81, 3684 (1984).

[b40] Stukowski A. Visualization and analysis of atomistic simulation data with OVITO–the Open Visualization Tool. Modelling Simul. Mater. Sci. Eng. 18, 015012 (2010).

