# Supplementary material for: Dislocation-enhanced experimental-scale vacancy loop formation in hcp Zirconium in one single collision cascade
Source: Sci Rep. 2016 Feb 12;6:21034. doi: 10.1038/srep21034 (PMC4751535; doi:10.1038/srep21034)
Supplement: Supplementary Information [file srep21034-s2.doc]

Supplementary Material For

**Dislocation-enhanced experimental-scale vacancy loop formation in hcp Zirconium in one single collision cascade**

Wei Zhou^a^, Jiting Tian^b^, Jian Zheng^a^, Jianming Xue^b,c,*^, Shuming Peng^a,*^

^a^Institute of Nuclear Physics and Chemistry, China Academy of Engineering Physics, Mianyang, Peoples Republic of China, 621999

^b^State Key Laboratory of Nuclear Physics and Technology, Department of Technical Physics, Peking University, Beijing, People’s Republic of China, 100871

^c^Center for Applied Physics and Technology, Peking University, Beijing, People’s Republic of China, 100871

^*^Corresponding author: jmxue@pku.edu.cn (Jianming Xue)

This Supplementary Material contains:

1 - Figures S1-S3

2 - Movie S1 (caption)

**1 - Supplementary Figures**


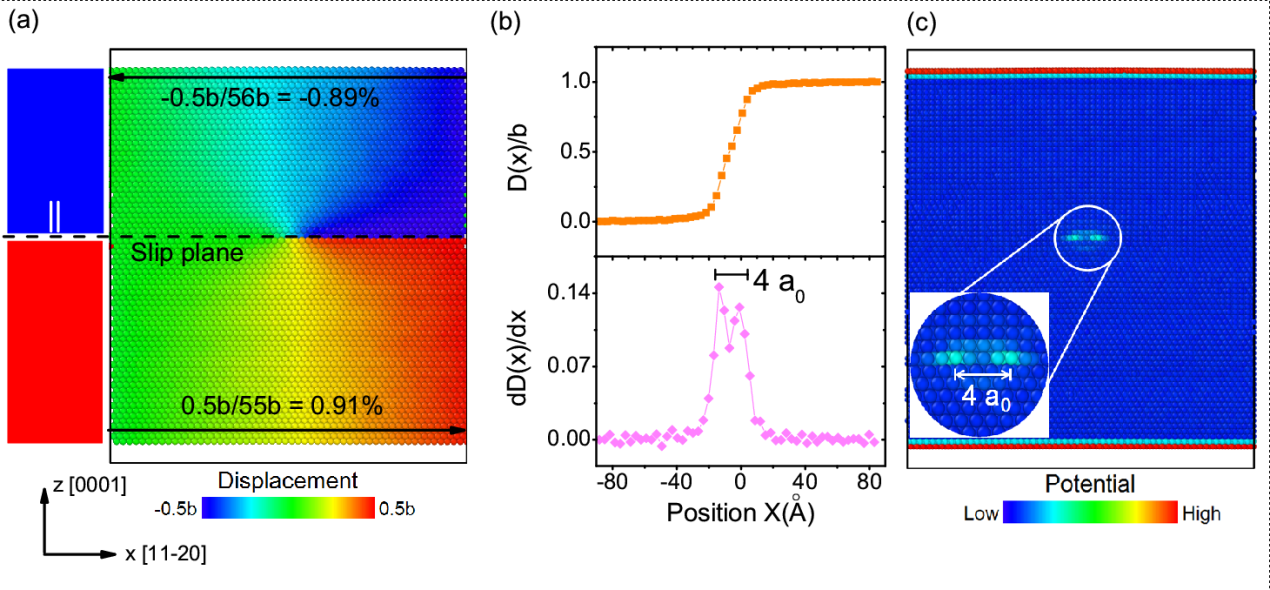


**Figure S1**: Simulated model system of the 1/3[11-20]{0001} ED in hcp Zr. (a) Displacement field of the sample after relaxation and quenching. (b) Disregistry D(x) around the dislocation core and the corresponding dislocation density dD(x)/dx. (c) Potential map of the sample after relaxation and quenching. See the Method section for details.


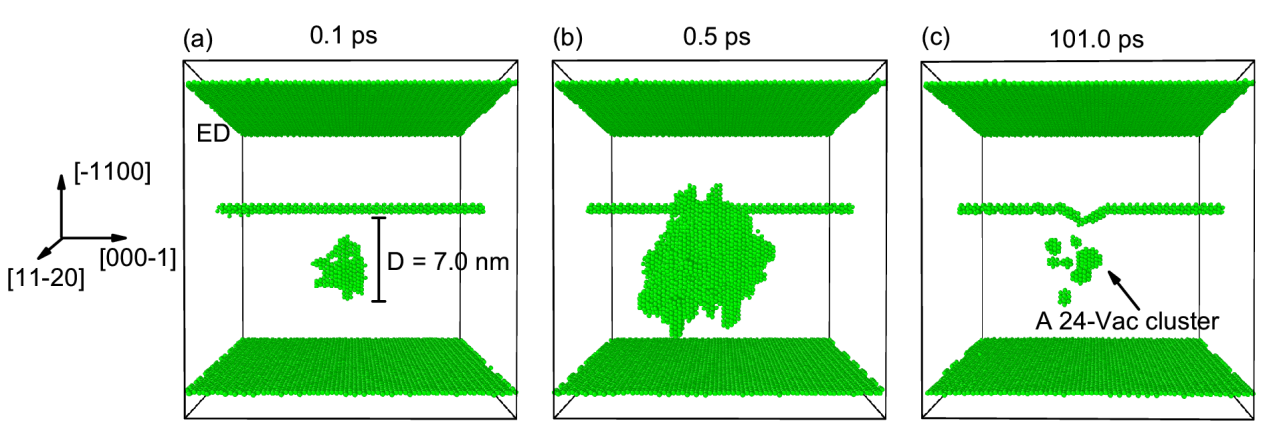


**Figure S2**: Temporal evolution of a typical cluster event induced by a 5keV PKA at D=7.0nm away from an 1/3[11-20]{-1100} ED in hcp Zr at 300K. Similar with the simulations using an 1/3[11-20]{0001} ED, large vacancy clusters containing up to 24 vacancies have been formed when the cascade region just hangs on the ED. In contrast, without the existence of an 1/3[11-20]{-1100} ED, the largest vacancy cluster formed in the 5 keV cascades in the pristine crystal only contains 12 vacancies. These test simulations prove that the choice of the preferred slip plane has no significant effects on the results.


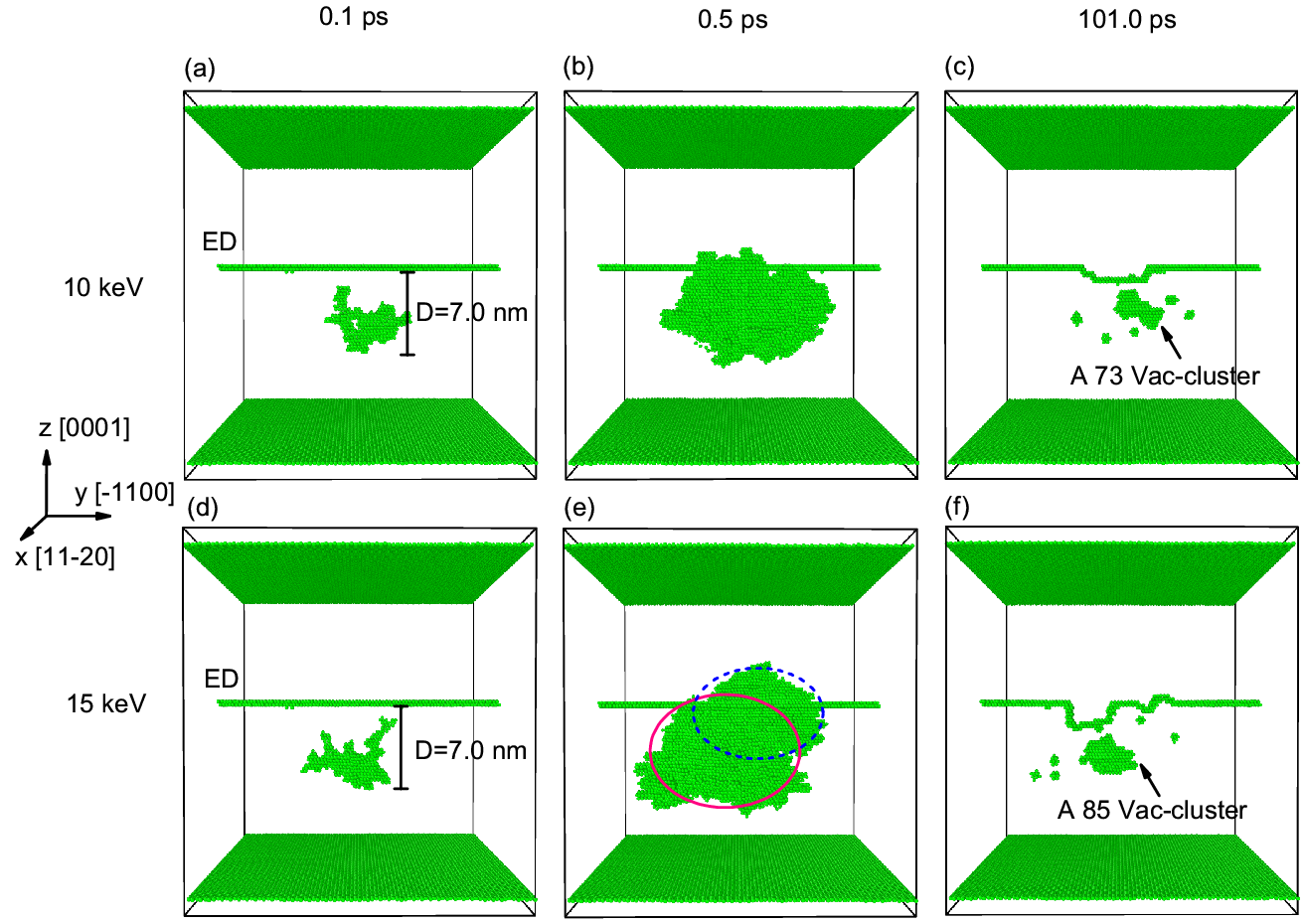


**Figure S3**: Temporal evolution of a typical cluster event induced by a 10 keV (top) or 15 keV (bottom) PKA in hcp Zr at 300K. In both cases, large vacancy clusters have been formed accompanied with the climb behavior of the ED. In the 15 keV event, a subcascade (dashed blue oval in (e)) is formed. The cluster is created by the first-order cascade (solid red oval in (e)) which just “hangs” on the ED. A more distinct separation between the first-order cascade and the subcascade is shown in the Supplementary Movie S1.

**2 – Caption of Supplementary Movie**

**Movie S1**: Whole temporal evolution of the 15 keV cluster event corresponding to Supplementary Fig. S3(d) - (f). Some of the snapshots have been shown in Fig. S3. We rotate the sample for a suitable angle so that the separation between the first-order cascade and the subcascade can be more distinct.
